# Functional Exercise Capacity and Perceived Exertion in Patients with Empty Nose Syndrome

**DOI:** 10.3390/diagnostics14090885

**Published:** 2024-04-24

**Authors:** Wei-Te Hung, Ta-Jen Lee, Pei-Wen Wu, Chi-Che Huang, Po-Hung Chang, Chien-Chia Huang

**Affiliations:** 1 Department of Medical Education, Chang Gung Memorial Hospital, Taoyuan 33302, Taiwan; p0614@cgmh.org.tw; 2 Division of Rhinology, Department of Otolaryngology, Chang Gung Memorial Hospital, Linkou 33305, Taiwan; entlee@cgmh.org.tw (T.-J.L.); a9665@cgmh.org.tw (P.-W.W.); hcc3110@cgmh.org.tw (C.-C.H.); bc1766@gmail.com (P.-H.C.); 3 School of Medicine, Chang Gung University, Taoyuan 33302, Taiwan; 4 Department of Otolaryngology, Xiamen Chang Gung Hospital, Xiamen 361028, China

**Keywords:** empty nose syndrome, functional exercise capacity, pulmonary function, perceived exertion, surgical intervention

## Abstract

Empty nose syndrome (ENS) is a complex condition characterized by symptoms such as dyspnea, nasal discomfort, and emotional challenges. This study aimed to evaluate functional exercise capacity and perceived exertion in patients with ENS. Patients with ENS who presented with a range of severe symptoms were prospectively enrolled. Pulmonary function was evaluated using spirometry, and functional exercise capacity was measured via the 6 min walk test (6-MWT). Perceived exertion was quantified using the Borg scale, and cardiopulmonary function was evaluated by monitoring peripheral oxygen saturation (SpO_2_). These parameters were assessed before and after nasal reconstruction surgery. A total of 44 patients with ENS were enrolled and classified into mild-to-moderate (n = 20) and severe (n = 24) symptom groups. Spirometry results showed no significant differences before and after surgery in the entire cohort. Perceived exertion showed significant postoperative improvement (*p* = 0.006). The severe ENS symptom group experienced significant improvement in SpO_2_ (*p* = 0.013) and perceived exertion (*p* = 0.002) at the end of the 6-MWT after surgery. Surgical intervention significantly enhanced functional exercise capacity (*p* = 0.038) in patients with mild-to-moderate ENS symptoms. Surgical reconstruction positively affected perceived exertion and SpO_2_ at the end of the 6-MWT in patients with ENS. The severity of ENS symptoms, as assessed by SNOT-25 scores, influenced these outcomes. These findings underscore the potential benefits of surgical intervention for enhancing exercise tolerance and respiratory efficiency.

## 1. Introduction

Empty nose syndrome (ENS), initially identified by Eugene Kern in the 1990s, encompasses a wide array of symptoms, such as nasal dryness, sensations of suffocation, fatigue, anxiety, depression, sleep disturbances, reduced concentration, and excessive daytime sleepiness (EDS) [1,2,3,4]. These psychological burdens often decrease motivation for physical activities, impacting overall functionality and exercise tolerance [5]. EDS is significantly associated with psychiatric comorbidity and limitations in functional capacity [6]. Some researchers have linked sleep disturbances to an amplified perception of exertion during exercise, which is known to decrease submaximal performance [7,8].

The upper and lower airways function collaboratively as integral anatomical and functional units. Paradoxical nasal obstruction in ENS due to substantially low nasal resistance and turbulent airflow through the nasal cavity may potentially affect pulmonary function [9]. ENS disrupts nasal aerodynamics due to the absence of nasal turbinates and the disturbance of the essential balance crucial for effective respiration. Reduced nasal resistance may disrupt the equilibrium required for deep pulmonary inspiration, resulting in a sense of breathlessness. Intriguingly, individuals with ENS may not perceive airflow through their nasal cavities despite their lungs registering inspiration [10]. This contradictory information for the autonomic nervous system might alter breathing patterns. Patients with ENS often report lower airway symptoms resembling those seen in hyperventilation syndrome (HVS), including chronic or exertional dyspnea and shortness of breath, which affect the respiratory and cardiac systems due to physiological inappropriateness [11,12]. Studies suggest that respiratory rehabilitation shows promise in alleviating these symptoms [13].

Understanding the association between the upper and lower airways and the effect of the ENS on them is crucial; however, comprehensive research in this field is currently lacking. The sino-nasal outcome test-25 (SNOT-25) evaluates upper airway symptoms in patients with ENS but may not cover all dyspnea- or exertion-related symptoms during physical activities [14]. This limitation might restrict its comprehensive assessment in ENS cases. Conversely, the Borg rating of the perceived exertion scale measures perceived exertion during activity [15]. Spirometry assesses lower airway function by measuring parameters like forced expiratory volume, airflow rates, lung volumes, and the capacity of the lungs [16]. The 6 min walk test (6-MWT) is widely acknowledged as a valuable tool for assessing aerobic capacity, endurance, and oxygen saturation across various medical conditions. Unlike other exercise tests, the 6-MWT allows patients to determine the intensity of their effort, providing a realistic reflection of their functional capacity during daily activities [17]. Despite the widespread application of the 6-MWT in various clinical contexts, its specific role in evaluating the impact of the ENS on exercise capacity and functional status remains largely unexplored in the existing literature. Thus, this study aimed to investigate functional exercise capacity and perceived exertion, as well as the effects of submucosal Medpor implantation on patients with ENS.

## 2. Materials and Methods

### 2.1. Study Design and Patient Selection

After approval was obtained from the Institutional Review Board of Chang Gung Medical Foundation (IRB numbers: 201802147A3 and 201902001A3), this prospective study was conducted at the Department of Otolaryngology between September 2018 and April 2021. Patients diagnosed with ENS who received care at the department were consecutively recruited after informed consent was obtained. The inclusion criteria were candidates for surgery for ENS aged 20–70 years. Patients with a history of pulmonary, cardiovascular, myoarticular, neurological, or psychiatric diseases, as well as craniofacial anomalies and other sinonasal conditions, were excluded. The diagnosis of ENS was primarily based on clinical manifestations, the presence of paradoxical nasal obstruction, a wide nasal airway and loss of inferior turbinate tissue observed by nasal endoscopy, a history of previous procedures on the inferior turbinate, and a positive cotton test, as described in a previous study [18]. The alleviation of symptoms upon performing the cotton test further confirmed the ENS diagnosis. The SNOT-25 questionnaire is a comprehensive tool covering various domains, including rhinologic symptoms, extranasal rhinologic symptoms, ear/facial symptoms, sleep dysfunction, psychological dysfunction, and empty nose symptoms, with a higher score indicating a more pronounced disease burden associated with ENS [14]. Previous research has established a correlation between the severity of ENS symptoms and EDS. Specifically, an SNOT-25 score exceeding 69 has been identified as a significant predictor of EDS, potentially affecting an individual’s motivation for physical activity and altering perceived effort, cognitive functioning, and exercise capacity [19]. Patients with ENS were categorized into two subgroups based on their SNOT-25 scores: those experiencing severe symptoms (SNOT-25 scores > 69) and those with mild-to-moderate symptoms (SNOT-25 scores ≤ 69), particularly during the preoperative phase of ENS management.

### 2.2. Nasal Reconstruction Procedure

Patients in the ENS group underwent a surgical procedure involving the endoscopic-assisted submucosal implantation of Medpor, a porous high-density polyethylene material (Medpor; Porex Surgical, Inc., Newnan, GA, USA). To meet individual patient requirements, the Medpor implants were customized into smaller sections. Surgery was performed under general anesthesia, and a submucosal pocket was carefully crafted along the lateral nasal wall to accommodate the Medpor implant. The implant size was determined based on the surgeon’s expertise and the dimensions of the created mucosal pocket in the inferior meatus. Postoperatively, patients received regular follow-up care at the outpatient clinic, with weekly visits during the initial month and subsequent appointments scheduled at intervals of 4–12 weeks, depending on the progress of their nasal recovery. In addition to surgical intervention, patients were instructed to use warm nasal saline irrigation 14 days after surgery.

### 2.3. Outcome Measures

Baseline and postoperative 3-month evaluations were also performed. Spirometric measurements, including forced vital capacity (FVC), forced expiratory volume in 1 s (FEV1), and the FEV1/FVC ratio, were performed in a seated position using a commercially available spirometer and nose clip for accuracy. Patients were instructed to withhold short-acting bronchodilators for six hours and longer-acting bronchodilators for 12–24 h before testing. The predicted values for these spirometry measurements were calculated on the basis of age, sex, and height.

Before the 6-MWT, baseline measurements of resting dyspnea, heart rate, peripheral oxygen saturation (SpO_2_), and blood pressure were recorded. The 6-MWT was conducted outdoors during the day to minimize potential variations in temperature and circadian rhythms. Participants were advised to consume a light meal and refrain from strenuous physical activity for at least 2 h before the test. They were instructed to wear comfortable attire and walking shoes. The 6-MWT involved participants walking as fast as possible for 6 min along a flat 40 m corridor. Participants were allowed to pause and rest if needed. After the test, the same measurements taken during the resting period were recorded, including the 6 min walk distance (6-MWD), which reflects functional exercise capacity, and the number of stops made during the test. The predicted 6-MWD was calculated using established sex-specific formulae that considered age, sex, height, and weight. These predictive formulae are widely recognized tools for evaluating the functional status of various patient groups, including those with chronic obstructive pulmonary disease (COPD), heart failure, arthritis, and neuromuscular disorders [20].

Perceived exertion at the end of the 6-MWT and resting dyspnea were assessed using the modified Borg scale, which ranges from 0 (indicating complete rest) to 10 (reflecting maximum exertion). This assessment provided valuable insights into the participants’ subjective experiences of exertion during physical activity. This comprehensive evaluation aimed to assess changes in functional exercise capacity and perceived exertion before and after surgical intervention in patients with ENS.

### 2.4. Statistical Analyses

Descriptive statistics, including mean values and standard deviations (SDs), were used. Data normality was assessed using the Shapiro–Wilk test to guide the selection of appropriate statistical tests. The Mann–Whitney U test or Wilcoxon signed-rank test was used to analyze non-normally distributed data, whereas normally distributed data were evaluated using the *t*-test. Spearman’s correlation coefficient was used to examine the associations between variables in the presence of non-normally distributed data. Statistical significance was set at *p* < 0.05.

## 3. Results

### 3.1. Participant Characteristics

Forty-four patients with ENS were included in this study and categorized into two groups based on their SNOT-25 scores as follows: mild-to-moderate ENS symptoms (SNOT25 score < 69, n = 20) and severe ENS symptoms (SNOT25 score > 69, n = 24). Demographic characteristics, including age, sex distribution, height, weight, body mass index (BMI), and spirometry results, were comparable between the groups, with no statistically significant differences observed. The prevalence of comorbidities, such as smoking, secondhand smoke exposure, asthma, atopic dermatitis, and other medical conditions, showed a similar distribution in both subsets of ENS patients (Table 1).

### 3.2. Pulmonary Function Test

At both the baseline and the 3-month postoperative assessment, the comparison between the mild-to-moderate and severe ENS symptom groups revealed no statistically significant differences in forced vital capacity (FVC) and forced expiratory volume in 1 s (FEV1) (*p* > 0.05). Specifically, at the baseline, the mean FVC values were 3.43 ± 0.69 L for the mild-to-moderate group and 3.15 ± 0.84 L for the severe group, with a *p*-value of 0.24. Similarly, the mean FEV1 values at baseline were 2.71 ± 0.60 L for the mild-to-moderate group and 2.62 ± 0.73 L for the severe group, with a *p*-value of 0.68. At the 3-month postoperative assessment, the mean FVC values were 3.42 ± 0.67 L and 3.20 ± 0.70 L for the mild-to-moderate and severe groups, respectively (*p* = 0.29), while the mean FEV1 values were 2.68 ± 0.56 L and 2.63 ± 0.62 L for the two groups, respectively (*p* = 0.75).

Additionally, within each symptom severity group, surgical intervention did not induce significant changes in the spirometry measurements at both time points (*p* > 0.05). The mean values of FVC, FEV1, and the FEV1/FVC ratio remained relatively stable before and after surgery in both groups, suggesting that surgical intervention did not significantly impact respiratory function as assessed by spirometry measurements in patients with mild-to-moderate or severe ENS symptoms.

### 3.3. Functional Exercise Capacity and Perceived Exertion

In the entire cohort of 44 patients with ENS, we observed a statistically significant reduction in perceived exertion with a decreased Borg rating scale score (*p* = 0.006) and an increased SpO_2_ at the end of the 6-MWT after surgery (*p* = 0.010) (Table 2). However, there was no difference in the perioperative evaluation of functional exercise capacity measured using the 6-MWD.

The correlation analysis revealed the perioperative questionnaire evaluations using the SNOT-25 and ENS6Q were significantly associated with the results Borg scale at the end of the 6-MWT (Table 3).

In the comparison between the mild-to-moderate and severe ENS symptom groups, there was a significant increase in the ratio of 6-MWD to predicted value (6-MWD/Predict) in the mild-to-moderate ENS symptom group, indicating improved functional exercise capacity after surgery (*p* = 0.038) (Table 4). Specifically, the mean 6-MWD for this group increased from 543.6 ± 81.1 m before the operation to 568.0 ± 66.9 m after the operation (*p* = 0.045), highlighting the significant postoperative improvement in functional exercise capacity.

**Figure 1:**
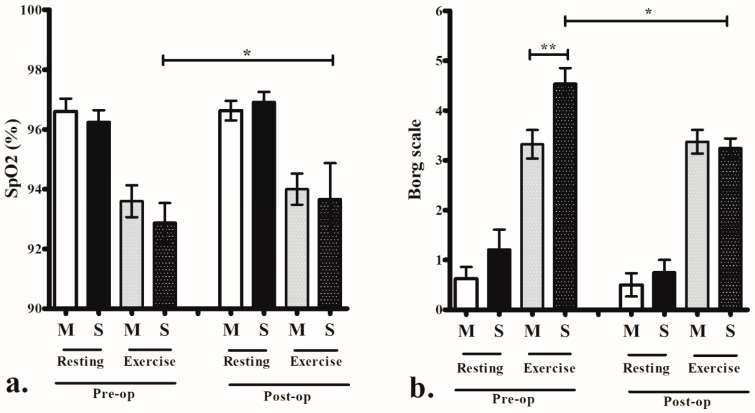
There was no difference in peripheral oxygen saturation (SpO_2_) (**a**) and perceived exertion by the Borg scale (**b**) between mild to moderate (M) and severe (S) empty nose syndrome (ENS) symptom groups at resting status before and after surgery. Patients with severe ENS symptoms experienced significant improvement in SpO_2_ (**a**) and perceived exertion (**b**) after surgery, achieving a similar level of perceived exertion in the mild to moderate ENS symptom group postoperatively (**b**). Data presented as mean ± standard error of mean. Preop, preoperative value; postop, postoperative value. * *p* < 0.05, ** *p* < 0.01.

At rest, we did not observe significant differences in perceived exertion measured using the Borg scale within or between the groups both before and at the 3-month postoperative evaluation. However, at the end of the 6-MWT, significant differences in perceived exertion according to the Borg scale score were noted between the groups preoperatively (*p* = 0.013). Following surgical intervention, the severe ENS symptom group exhibited remarkable improvements in perceived exertion on the Borg scale (*p* = 0.002), reaching levels comparable to those of the mild-to-moderate ENS symptom group. The resting, end, and peak heart rates measured at the beginning and end of the test did not show significant differences between the groups. However, the severe ENS symptom group also exhibited a significant improvement in SpO_2_ at the end of the 6-MWT (*p* = 0.013) and the 3-month postoperative evaluation (Table 4) (Figure 1).

## 4. Discussion

In this study, we aimed to investigate the potential effects of the severity of ENS symptoms, categorized based on SNOT-25 scores as severe (SNOT25 score > 69) and mild-to-moderate (SNOT25 score < 69) on impairments of functional exercise capacity and the altered quality of life in affected individuals [21]. We introduced the 6-MWT and Borg scale to evaluate the functional exercise capacity and subjective perception of breathlessness during submaximal exercise [22]. While the SNOT-25 questionnaire primarily assesses subjective nasal symptoms and associated quality of life, we recognized that the subjective perception of breathlessness at the baseline before the 6-MWT shares similarities with the evaluation of obstructive nasal symptoms included in the SNOT-25 questionnaire [23,24]. Compared to previous studies that mostly relied on the SNOT-25 and ENS6Q questionnaires to assess patients’ subjective symptoms during daily activities, this study gained a comprehensive understanding of the impact of ENS on patients’ ability to perform physical activities and their subjective experience of breathlessness during submaximal exercise by combining objective measures of functional exercise capacity with subjective perceptions.

The Borg scale, known for its cost-effectiveness and practicality, is renowned for its efficacy in assessing disease burden and improving various respiratory conditions [25,26]. Previous research has highlighted that even minor alterations in the Borg breathlessness scale scores, particularly at comparable exercise levels, can have clinically significant implications, particularly for patients with chronic lung conditions undergoing pulmonary rehabilitation or exercise interventions [27]. Observations from Table 2 indicate that while Borg scale scores at End 6MWT significantly decreased between preoperative and postoperative assessments, the changes between End 6MWT and resting were not significantly different between the two groups. Moreover, it is important to note that the absolute change in Borg scale values may not necessarily represent an equivalent degree of change in perceived breathlessness. The numerical differences between Borg scale ratings do not always reflect linear changes in perceived exertion. Therefore, while variations in Borg scale ratings offer insights into the relative changes in breathlessness, they may not fully capture the nuanced nature of perceived exertion.

Spirometry results demonstrated no significant differences between the severe and mild-to-moderate symptom groups before and after surgery, suggesting that most ENS patients may not experience apparent activity limitations during static activities. While traditional pulmonary function tests play indispensable roles in evaluating cardiorespiratory health, they may not fully elucidate the specific changes and limitations experienced by individuals with ENS during physical activity [23]. Patients with congestive heart failure, restrictive lung disease, or COPD may not perceive discomfort during low physical activities due to the lower demands placed on their cardiovascular and respiratory systems. However, when confronted with increased exercise intensity, the combined effects of exercise-induced hypoxemia, ventilatory and cardiac limitations, muscle weakness, gas exchange inefficiency, and airway reactivity may render patients unable to sustain the increased workload, leading to exercise intolerance and discomfort [28,29,30]. Proper exercise testing and individualized exercise programs can help identify and manage these limitations in individuals with ENS, ultimately improving their overall exercise tolerance and quality of life.

In our study of 44 patients, surgery significantly improved perceived exertion. Further analysis showed a correlation between the severity of ENS symptoms, as measured by the SNOT-25 questionnaire and its different domains, and perceived physical exertion. Specifically, the SNOT-25 scores and the sleep dysfunction domain exhibited moderate positive correlations with the End 6MWT Borg scale, indicating that higher sinonasal symptom levels and sleep dysfunction are associated with increased perceived exertion during the 6MWT. This suggests that individuals with more severe ENS symptoms and greater sleep disturbances may experience heightened breathlessness during physical activity. We categorized severity into two groups using the SNOT-25 questionnaire, finding that individuals with severe ENS symptoms experienced elevated breathlessness perception during exercise compared to those with mild-to-moderate symptoms. This suggests that severity assessed using the SNOT-25 questionnaire may reflect the sensitivity of perceived exertion during physical activity.

Our study aligns with previous research indicating that surgical intervention can enhance the nasal sensation of airflow during daily activities [31]. Importantly, our study extends this understanding by showing that surgical intervention can reduce perceived exertion during exercise, especially among patients with more severe symptoms (Figure 1). These findings underscore the importance of considering self-perceived exertion symptoms during submaximal exercise as novel indicators for assessing ENS symptom severity and treatment outcomes.

Furthermore, our study utilized pulse oximetry [32], a non-invasive test, to monitor blood oxygen levels, which are particularly valuable for identifying breathing issues in individuals with sleep disorders [33]. Exercise-induced desaturation, common in those with compromised respiratory function, results in decreased SpO_2_ levels. This triggers compensatory mechanisms such as increased respiratory and heart rates, leading to feelings of breathlessness and heightened effort, which are often measured using the Borg scale [34].

Enhancements in SpO_2_ levels and perceived exertion postsurgery suggest potential improvements in cardiopulmonary function. Surgery may reduce exercise-induced desaturation by optimizing nasal airflow and oxygen delivery and lessening the need for increased respiratory and heart rates during physical activity. This could alleviate breathlessness and perceived effort, ultimately enhancing exercise tolerance and quality of life in ENS patients.

Our findings indicate that patients with milder ENS symptoms experience substantial improvements in functional exercise capacity after surgery, which could be attributed to the positive impact of surgical intervention on psychological well-being. Improvements in emotional well-being after surgery may contribute to a reduction in perceived physical exertion. Individuals with chronic respiratory conditions often develop a pattern of avoiding activities that trigger dyspnea, known as the “fear of dyspnea” cycle [35]. This cycle persists as individuals gradually curtail their physical activity levels to alleviate the distressing sensation of breathlessness, frequently accompanied by feelings of fear and anxiety. Previous research indicated that nasal reconstruction surgery may facilitate the restoration of a more natural airflow pattern in the nasal passage, potentially realigning the disrupted balance of resistance essential for deep pulmonary inspiration [36,37,38]. Consequently, it may alleviate the strain on the respiratory muscles, resulting in a reduced perception of exertion during exercise.

The intricate interplay between psychological factors and exercise capacity is a phenomenon observed in various medical conditions [39]. For instance, in patients with COPD, depression has been linked to decreased performance in the 6-MWT [40,41]. Similarly, ENS patients with high SNOT-25 scores often experience depression and anxiety, which are factors that can influence their exercise performance. Surgical treatment has proven effective in alleviating depression and anxiety symptoms in ENS patients [3]. When perceived exertion is reduced through surgical intervention, individuals may experience less discomfort and greater confidence in their ability to engage in daily activities, thereby boosting their motivation to be physically active.

Chronic or exertional breathlessness is often associated with hyperventilation syndrome (HVS) in patients with ENS [13]. HVS can lead to decreased carbon dioxide (CO_2_) levels in the blood and respiratory alkalosis. This imbalance has widespread physiological effects, influencing cardiovascular function and respiratory mechanics and ultimately affecting exercise capacity [42]. Reduced blood CO_2_ levels can lead to vasoconstriction, diminishing oxygen delivery to the tissues, including respiratory muscles [43]. Respiratory muscle fatigue and weakness may occur, further compromising functional exercise capacity in individuals with ENS and HVS [44]. In the context of ENS, the reduction in turbinate tissue often results in enlarged airspace and disrupted airflow dynamics, contributing to recurrent dyspnea and a heightened urge to take deep breaths. Over time, these altered breathing patterns can further affect the respiratory muscles, potentially leading to respiratory muscle dysfunction. This dysfunction may manifest as reduced inspiratory muscle strength and endurance, which is a phenomenon that has been extensively studied in patients with COPD [45].

Following nasal cavity reconstruction, which narrows the airway and increases air resistance, respiratory patterns may improve, enhancing gas exchange and oxygen transport to the peripheral muscles during physical exertion, thus boosting muscle strength and efficiency [31]. However, individuals with severe ENS symptoms may struggle to adjust their breathing patterns, often limiting physical activities due to breathlessness and discomfort, potentially resulting in a lower baseline exercise capacity that makes immediate improvements less noticeable.

To assess cardiopulmonary function in patients with ENS, future studies should consider employing respiratory muscle strength testing (RMST) and cardiopulmonary exercise testing (CPET). RMST evaluates respiratory muscle fatigue, which is crucial in conditions like obstructive sleep apnea (OSA) and COPD [46], while CPET provides insights into cardiovascular, respiratory, and metabolic responses during physical activity [47]. Utilizing CPET can offer objective measures of exercise tolerance, aerobic capacity, and ventilatory responses, especially given the shared symptoms between ENS patients and those with OSA [48].

Integrating preoperative and postoperative rehabilitation programs holds promise for enhancing the exercise capacity of patients with ENS. A structured regimen combining aerobic and resistance training [49], along with specific breathing exercises, can improve lung function and reduce perceived exertion during exercise [50]. Personalized rehabilitation and psychological support are essential for addressing both the physical and psychological aspects of ENS management, enabling patients to actively engage in daily activities and enhance their quality of life. Further research into the effectiveness of cognitive-behavioral therapy and targeted mental health interventions is warranted to address associated anxiety and depression, ultimately leading to improved patient care and outcomes.

The prevalence and incidence of ENS remain poorly understood, making it a relatively rare clinical entity. Consequently, our study is limited by the rarity of ENS, which results in a limited pool of eligible participants. Ethical considerations prevented us from conducting a double-blind placebo-controlled study and random allocation to the treatment arms. Additionally, the presence of comorbidities posed challenges in recruiting patients exclusively affected by ENS in a tertiary care hospital setting. However, these challenges were mitigated through rigorous statistical analyses of the baseline data, which demonstrated comparability between the two study groups in terms of physical characteristics, smoking habits, clinical profiles, baseline spirometry, and 6-MWT indicators. It is important to note that the follow-up in our study was limited to the 3-month postoperative period, potentially limiting our understanding of long-term outcomes or changes over an extended duration.

Future research with larger sample sizes and longer follow-up durations is essential to validate observed improvements in empty nose syndrome (ENS) management. Incorporating additional outcome measures, such as respiratory muscle strength testing and cardiopulmonary exercise testing, could provide broader insights into ENS patients’ cardiopulmonary function.

## 5. Conclusions

Surgical intervention with submucosal Medpor implantation resulted in substantial improvements in perceived exertion and SpO_2_ at the end of the 6-MWT in patients with ENS, which were influenced by the severity of ENS symptoms, as assessed by SNOT-25 scores. These findings underscore the potential benefits of surgical intervention for enhancing exercise tolerance and respiratory efficiency.

## Figures and Tables

**Table 1:** Clinical characteristics of the study participants at baseline.

Characteristics	All Patients (n = 44)	Mild-to-Moderate (n = 20)	Severe (n = 24)	*p* Value ^†^
Age (year)	50.9 ± 12.1	51.1 ± 9.6	50.7 ± 14.1	0.914
Gender (male/female)	60:28	15:5	15:9	
Height (cm)	166.2 ± 7.9	167.7 ± 6.9	164.9 ± 8.4	0.255
Weight (kg)	67.2 ± 12.2	69.9 ± 12.1	64.9 ± 12.1	0.134
BMI (kg/m^2^)	24.3 ± 3.5	24.7 ± 3.5	23.8 ± 3.5	0.368
SNOT-25	67.2 ± 21.0	49.0 ± 15.2	82.4 ± 10.3	<0.001 ***
ENS6Q	14.3 ± 5.6	10.6 ± 3.1	17.4 ± 5.4	<0.001 ***
Spirometry				
FVC/predicted (%)	88.9 ± 12.6	89.6 ± 8.4	88.4 ± 15.5	0.770
FVE1/predicted (%)	87.5 ± 12.5	86.0 ± 10.7	88.8 ± 13.9	0.460
FEV1/FVC (%)	81.5 ± 7.2	78.7 ± 8.2	83 ± 5.9	0.051
Comorbidity				
Smoking	5	3	2	
Secondhand smoke exposure	5	3	2	
Asthma	3	1	2	
Atopic dermatitis	7	4	3	
Other else	14	4	10	

Data are presented as mean ± SD. Abbreviation: BMI, body mass index; SNOT-25, sino-nasal outcome test-25; ENS6Q, Empty Nose Syndrome 6-item Questionnaire. *** *p* < 0.001. ^†^ compare between mild-to-moderate symptom group and severe symptom group.

**Table 2:** Perioperative evaluations of functional exercise capacity and perceived exertion.

		Preoperative	Postoperative	*p* Value
Saturation (SpO_2_)	Resting	96.4 ± 1.9	96.9 ± 1.5	0.103
	End 6MWT	93.2 ± 2.9	94.2 ± 2.5	0.010 *
	Change (Δ)	−3.2 ± 0.4	−2.6 ± 2.3	0.095
Heartbeat (min)	Resting	82.2 ± 14.5	81.6 ± 13.0	0.745
	End 6MWT	109.7 ± 21.0	108.7 ± 23.3	0.777
Peak heart rate		123.2 ± 17.9	123.0 ± 17.5	0.941
Borg scale	Resting	0.9 ± 1.6	0.6 ± 1.1	0.386
	End 6MWT	4 ± 1.5	3.3 ± 1.0	0.006 **
	Change (Δ)	3.0 ± 1.8	2.7 ± 1.1	0.169
6MWD (m)		546.5 ± 66.8	547.1 ± 65.8	0.889
Predicted 6MWD (m)		587.9 ± 84.4		
6MWD/predict (%)		94.2 ± 14.1	94 ± 11.7	0.915

Data are presented as mean ± SD. 6MWT, 6-Minute Walk Test; 6MWD, 6 min walking distance; Resting; Change (Δ), Change in 6MWT = End 6MWT—Resting; * *p* < 0.05; ** *p* < 0.01.

**Table 3:** Correlation analysis between empty nose symptom evaluations and End 6MWT Borg scale.

	Spearman’s R (95% CI)	*p* Value
SNOT-25	0.309 (0.104~0.479)	0.003 **
Sleep dysfunction domain	0.305 (0.103~0.475)	0.004 **
ENS domain	0.247 (0.041~0.436)	0.020 *
Psychological dysfunction domain	0.242 (0.029~0.438)	0.023 *
ENS6Q	0.219 (−0.002~0.412)	0.041 *

SNOT-25, Sino nasal outcome test-25; ENS, empty nose syndrome; ENS6Q, Empty Nose Syndrome 6-item Questionnaire. * *p* < 0.05; ** *p* < 0.01.

**Table 4:** Perioperative evaluations of functional exercise capacity and perceived exertion in two groups.

Parameters		Mild to Moderate Group (n = 20)			Severe Group (n = 24)		
		Preop	Postop		Preop	Postop	
		Mean ± SD	Mean ± SD	*p* Value	Mean ± SD	Mean ± SD	*p* Value
Saturation	Resting	96.6 ± 1.9	96.8 ± 1.5	0.809	96.3 ± 1.9	97.0 ± 1.6	0.057
	End 6MWT	93.6 ± 5.6	94.0 ± 2.3	0.355	92.9 ± 3.2	94.4 ± 2.8	0.013 *
	Change (Δ)	−3 ± 2.3	−2.8 ± 2.0	0.614	−3.4 ± 3.0	−2.5 ± 2.5	0.054
Heart Beat	Resting	83.5 ± 13.4	81.2 ± 10.7	0.210	83.0 ± 15.6	82.0 ± 14.9	0.563
	End 6MWT	109.6 ± 19.0	106.7 ± 15.2	0.493	109.8 ± 23.2	110.4 ± 28.7	0.692
Peak Heart Rate		119.7 ± 14.1	123.7 ± 16.8	0.171	126.0 ± 19.0	122.4 ± 18.3	0.336
Borg scale	Resting	0.6 ± 1.0	0.5 ± 1.1	0.610	1.2 ± 2.0	0.8 ± 1.2	0.437
	End 6MWT	3.3 ± 1.3	3.4 ± 1.1	0.724	4.5 ± 1.5	3.3 ± 0.9	0.002 **
	Change (Δ)	2.7 ± 1.3	2.9 ± 1.1	0.908	3.3 ± 2.1	2.5 ± 1.1	0.078
6MWD (m)		543.6 ± 81.1	568.0 ± 66.9	0.045 *	549.0 ± 53.8	529.8 ± 69.8	0.115
6MWD/Predict (%)		92.8 ± 12.6	96.9 ± 9.9	0.038 *	95.4 ± 15.5	91.6 ± 12.8	0.103

Preop, preoperative; postop postoperative; 6MWT, 6-Minute Walk Test; 6MWD, 6 min walking distance * *p* < 0.05; ** *p* < 0.01.

## Data Availability

The dataset used and/or analyzed during the current study is available from the corresponding author upon reasonable request.
